# Robust Depth Image Acquisition Using Modulated Pattern Projection and Probabilistic Graphical Models

**DOI:** 10.3390/s16101740

**Published:** 2016-10-19

**Authors:** Jaka Kravanja, Mario Žganec, Jerneja Žganec-Gros, Simon Dobrišek, Vitomir Štruc

**Affiliations:** 1 Alpineon d.o.o., Ulica Iga Grudna 15, Ljubljana SI-1000, Slovenia; jaka.kravanja@alpineon.si (J.K.); mario.zganec@alpineon.si (M.Ž.); jerneja.gros@alpineon.si (J.Ž.-G.); 2 Faculty of Electrical Engineering, University of Ljubljana, Tržaška cesta 25, Ljubljana SI-1000, Slovenia; simon.dobrisek@fe.uni-lj.si

**Keywords:** depth imaging, modulated acquisition, structured light, triangulation, probabilistic graphical models, 3D reconstruction

## Abstract

Depth image acquisition with structured light approaches in outdoor environments is a challenging problem due to external factors, such as ambient sunlight, which commonly affect the acquisition procedure. This paper presents a novel structured light sensor designed specifically for operation in outdoor environments. The sensor exploits a modulated sequence of structured light projected onto the target scene to counteract environmental factors and estimate a spatial distortion map in a robust manner. The correspondence between the projected pattern and the estimated distortion map is then established using a probabilistic framework based on graphical models. Finally, the depth image of the target scene is reconstructed using a number of reference frames recorded during the calibration process. We evaluate the proposed sensor on experimental data in indoor and outdoor environments and present comparative experiments with other existing methods, as well as commercial sensors.

## 1. Introduction

Over the last few years, we have witnessed the rapid growth of 3D imaging technologies in various application areas, ranging from autonomous navigation of robots, drones or cars [1,2,3], medical applications [4,5], consumer electronics [6] and surveillance systems [7,8] to object reconstruction [9,10], biometrics [11,12,13], and others [14,15,16]. Especially, with the introduction of low-cost commercial 3D imaging sensors, such as Microsoft’s Kinect (Microsoft, Redmond, WA, USA) [6], depth sensing has become a popular research direction with new applications and use cases being presented on a regular basis. Several major corporations have since introduced their own depth imaging technology (e.g., Intel (Santa Clara, CA, USA) recently announced the Euclid sensor; Sony (Tokio, Japan) introduced the PlayStation Camera with the PS4 console; and Infineon (Neubiberg, Germany) developed the Real3 sensor) with the goal of participating in this rapidly growing depth-sensor market.

Existing 3D imaging techniques can be divided into two main categories: (i) active and (ii) passive. Active techniques utilize an active source of illumination to project a suitably-devised pattern of structured light onto the target scene and then perform 3D reconstruction based on temporal or spatial distortions of the projected pattern caused by interactions with the target scene. Examples of active techniques include standard structured light approaches [17,18,19], time-of-flight methods [20,21] or interferometry [22]. A comprehensive review of existing techniques from this group can be found in [23,24]. Passive techniques, on the other hand, do not rely on active illumination, but commonly require only a calibrated pair of cameras. Typical examples of passive techniques represent stereo-vision [25], shape-from-focus [26], shape-from-shading [27] and other related shape-from-X approaches [28]. The reader is referred to [29] for more detailed coverage of this topic.

When applied in outdoor environments, 3D imaging techniques are expected to provide accurate depth information regardless of the external lighting and atmospheric conditions, which are known to negatively affect the existing range measurement techniques. This property is crucial for various outdoor applications that require reliable depth information to function properly. This paper addresses the problem of outdoor depth imaging with structured light approaches and presents a novel sensor designed specifically for outdoor deployment [30]. The sensor exploits the recently-proposed concept of modulated pattern projection, introduced by our group in [31], which facilitates the acquisition of spatial distortion maps in real-world environments (even in the presence of strong incident sunlight), where other existing approaches often fail or at least struggle with their performance. Correspondences between the projected pattern and the acquired distortion map are established based on a novel probabilistic approach relying on graphical models (inspired by [32,33]) and are used in conjunction with prerecorded reference frames to compute the depth information for each point of the projected structured light pattern. All components of the sensor presented are designed for outdoor deployment and contribute to the overall performance, as demonstrated in the experimental section.

We make the following three contributions in this paper: (i) we present a novel 3D imaging sensor that supports robust acquisition of spatial distortion maps and is able to generate accurate depth maps in challenging outdoor environments; (ii) we describe the complete hardware and software (algorithmic) design of the sensor; and (iii) we present a comprehensive experimental evaluation, as well as comparative results with competing techniques and existing commercial sensors.

The paper is organized as follows: Section 2 presents the main components of the depth sensor and outlines their characteristics. The individual components are discussed in Section 3, Section 4 and Section 5. Experimental results and comparative evaluations are described in detail in Section 6. The paper concludes with some final remarks and directions for future work in Section 7.

## 2. Sensor Overview

This section presents a short overview of a novel sensor designed for depth image acquisition. The sensor presented was developed as part of our research efforts with respect to an active triangulation system (ATRIS) capable of capturing depth images in difficult settings; for example, under exposure to strong incident sunlight. The sensor relies on the established concept of depth image acquisition based on structured light, in which a light pattern is first projected onto a target scene, and the shape (i.e., depth information) of the scene is then inferred based on the spatial distortions of the projected pattern and the (known) geometrical properties of the prototype.

**Figure 1:**
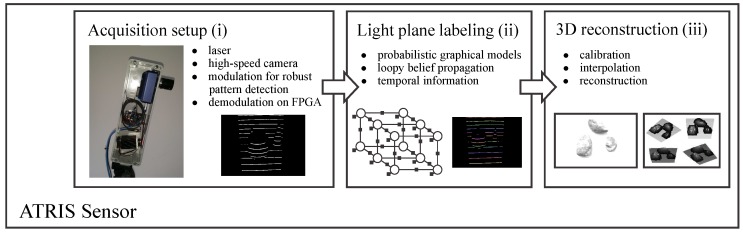
Schematic representation of our active triangulation system (ATRIS) sensor. The sensor comprises three key components: (i) an image acquisition procedure, which captures an image of the projected light pattern; (ii) a light plane labeling technique, which establishes the correspondence between all parts of the projected and detected patterns; and (iii) a 3D reconstruction procedure, which constructs a depth image from the detected light pattern.

A schematic representation of the three key components of the ATRIS sensor is shown in Figure 1.

- The image acquisition procedure uses specialized hardware (comprised of a laser projector and a high-speed camera) to project a structured light pattern onto the target scene with the goal of capturing an image of the distorted pattern (i.e., a spatial distortion map). The procedure is based on the recently-introduced concept of modulated pattern projection [31,34], which ensures that spatial distortion maps of good quality can be captured in challenging conditions; for example, in the presence of strong incident sunlight or under mutual interference caused by other similar sensors directed at the same scene.
- The light plane-labeling procedure establishes the correspondence between all parts of the projected light pattern and the detected pattern that has been distorted due to the interaction with the target scene. The procedure uses loopy-belief-propagation inference over probabilistic graphical models (PGMs) as proposed in [33] to solve the correspondence problem and, differently from other existing techniques in the literature, exploits spatial relationships between parts of the projected pattern, as well as temporal information from several consecutive frames to establish correspondence.
- The 3D reconstruction procedure reconstructs the depth image of the target scene based on (i) the reference frames of the light pattern projected onto a planar surface at different distances from the camera and (ii) the established correspondence between parts of the projected pattern and the detected distortion map.

A detailed description of all three key ATRIS components is presented below.

## 3. The Acquisition Procedure

This section describes the modulated pattern acquisition procedure used in the ATRIS sensor. The section starts by presenting the hardware setup used in the sensor and then proceeds by describing the pattern acquisition procedure and its characteristics. Note that the underlying concept of the acquisition procedure was originally introduced in [31].

**Figure 2:**
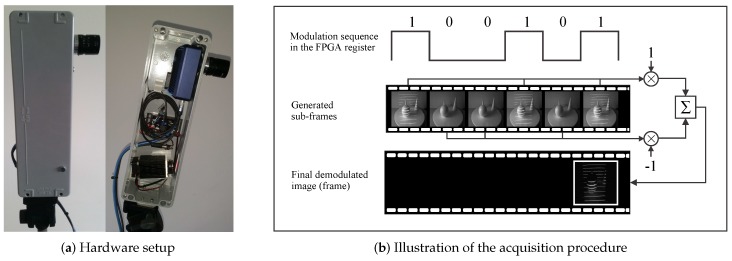
The acquisition procedure. (**a**) Visual appearance of the hardware setup of the ATRIS sensor. The left side of the image shows the casing of the sensor and the right side the arrangement of the camera (above) and the laser projector (below) in the casing. (**b**) Illustration of the modulated acquisition procedure.

The hardware setup (shown in Figure 2a) used in the ATRIS sensor comprises a high-speed industrial camera with an integrated FPGA processing core and a modulable 650-nm LED laser. The high-speed (Velociraptor [35]) camera (Optomotive, Ljubljana, Slovenia) is capable of operating at a frame rate of 480 fps, which allows the ATRIS sensor to capture images of the structured light pattern at a speed of several frames per second. The sensor can therefore be used with static or dynamic scenes.

To acquire one image (or better said, a single frame) of the projected light pattern, the camera first captures a series of images of the target scene (we refer to these images as sub-frames). Every time an image (sub-frame) is taken, the laser projector is turned either on or off depending on the current value of the pseudo-random binary control/modulation sequence c∈{0,1} that is cyclically shifted in the FPGA modulation register [31]. If the control/modulation sequence takes a value of c=1, the laser projector is turned on, and the captured sub-frame contains a snapshot of the illuminated target scene. Similarly, when the control/modulation sequence takes a value of c=0, the laser projector is turned off, and the captured sub-frame contains a snapshot of the scene without the structured light pattern (see Figure 2b). Based on these sub-frames, the final image of the projected pattern is generated as a normalized superposition of all sub-frames captured during one cycle of the control sequence.

As illustrated in Figure 2b, all sub-frames captured during the on state of the laser are added to the superposition, and all sub-frames captured during the off state are subtracted. This demodulation procedure is implemented in FPGA and removes most information about the appearance of the target scene from the generated image/frame, thus significantly emphasizing the projected pattern. A thresholding step is ultimately applied to the demodulated image to remove all remaining (scene-related) artifacts and to produce the final binary image of the projected pattern needed for the light plane labeling.

The acquisition procedure presented exhibits several desirable characteristics that are also experimentally validated in Section 6.1 (for a formal theoretical argumentation of the characteristics, the reader is referred to [31]):

- Noise suppression: The modulated acquisition procedure is robust for various types of noise. If information related to the visual appearance of the target scene is treated as “background noise,” then the procedure presented obviously removes the background noise as long as the control/modulation sequence employed in the FPGA register is balanced (a balanced modulation sequence is defined as a sequence with an equal number of zero- and one-valued bits). Because demodulation is a pixel-wise operation, the acquisition procedure presented also suppresses sensor noise (typically assumed to be Gaussian) caused, for instance, by poor illumination or high temperatures, where a simple pair-wise sub-frame subtraction would not suffice.
- Operation under exposure to incident sunlight: Even if the illumination of the target scene by incident sunlight is relatively strong, the modulation sequence is capable of raising the level of “signal” pixels sufficiently to recover a good-quality image of the projected pattern. This characteristic is related to the noise suppression property discussed above, because incident sunlight behaves very much like background noise under the assumption that the intensity level of the sunlight is reasonably stable.
- Mutual interference compensation: With the modulated acquisition procedure, it is possible to compensate for the mutual interference typically encountered when two or more similar sensors operate on the same target scene. This can be done by constructing the control/modulation sequences based on cyclic orthogonal (Walsh–Hadamard) codes, in which the cross-correlation properties of the modulation codes are exploited to compensate the mutual interference (see [31] for more information). Similar concepts are used in other areas, as well; for example, for synchronized CDMA (code division multiple access) systems [36] or sensor networks [37], for which mutual interference also represents a major problem.

**Figure 3:**
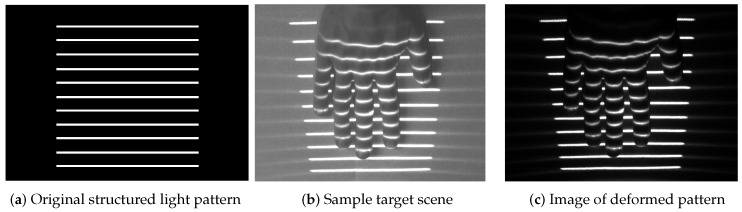
The correspondence problem: (**a**) an illustrative image of the structured light pattern produced by the ATRIS sensor, without distortions; (**b**) an example of a target scene illuminated by the light pattern; (**c**) an example of the spatial distortion map captured with the sensor. To be able to reconstruct a depth image of the target scene, each pixel comprising the light pattern in (**c**) needs to be assigned a label corresponding to one of the light planes in the pattern shown in (**a**).

It should be noted that in the current implementation of the ATRIS sensor, a diffractive optical element (DOE) mounted in front of the laser is used to split the laser beam and produce a structured light pattern comprising 11 parallel light planes (see Figure 3a). For the modulation sequence, a 16-bit long modulation sequence is used, which results in a stable pattern acquisition rate of 30 fps given a camera frame rate of 480 fps (i.e., 480 fps/16 = 30 fps).

## 4. Light-Plane Labeling

The modulated acquisition procedure presented in the previous section results in a binary image (or frame) of the projected pattern that forms the basis for depth image reconstruction. As pointed out in Section 2, the depth image is computed based on the geometrical properties of the acquisition setup and the distortions of the structured light pattern caused by projecting the light pattern onto the target scene [33]. Although the geometrical properties of the acquisition setup are commonly known in advance, the pattern distortions need to be quantified before a depth image can be constructed. Typically, this is achieved by establishing the correspondence between all parts of the original structured light pattern (Figure 3a) and all parts of the captured distortion map (Figure 3c). Because solving this correspondence problem (illustrated in Figure 3) is crucial for the success of the depth-image-construction step, an efficient procedure based on probabilistic graphical models (PGMs) was developed for the ATRIS sensor. A detailed description of the procedure is given below in this section.

### 4.1. Problem Statement

**Figure 4:**
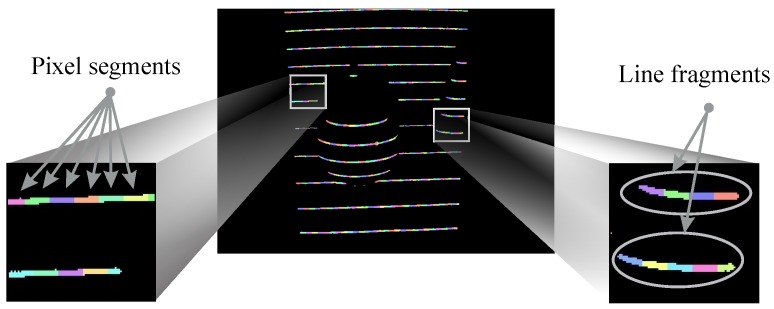
Visual illustration of some terminology used in this paper. Connected binary regions (using eight-adjacency) are referred to as line fragments. Smaller parts of the line fragments of fixed width are referred to as pixel segments.

The binary distortion map captured with the ATRIS acquisition setup contains a large number of binary regions. The 11 light planes that constitute the structured light pattern are usually not detected as large connected binary regions in the captured image, but in the form of shorter, potentially discontinuous line fragments (we refer to any connected binary region (using eight-adjacency) in the image as a line fragment), as shown in Figure 4. In addition, small binary regions not corresponding to any of the projected light planes can also appear in the captured image due to the presence of noise.

The structured light pattern used in the ATRIS sensor consists of 11 parallel light planes. Solving the correspondence problem, therefore, amounts to finding the correct light plane label for each of the connected binary regions in the captured binary distortion map [33]. Although this labeling problem could be approached for each non-zero pixel individually, we group the non-zero pixels into pixel segments (i.e., parts of the line fragments with a fixed width; see Figure 4) and try to assign each pixel segment one of the light plane labels to reduce the computational burden of the labeling procedure.

The illustrated labeling problem can be formally defined as follows: assume that the detected light pattern is represented in the form of the binary distortion map *I*, that the scene points illuminated by the projected pattern are encoded with a pixel value of one and that all other pixels are encoded with a value of zero. Furthermore, assume that the non-zero pixels that form the line fragments are grouped into pixel segments of fixed width (i.e., spanning a predefined number of image columns). Let us now denote the set of all pixel segments in the distortion map *I* as $P=\{p_{1},p_{2},\ldots ,p_{N}\}$, where $p_{i}$ stands for the *i*-th pixel segment (for i=1,2,...,N) and *N* represents the number of all pixel segments in *I*. Moreover, let us denote the set of indices of the light planes constituting our pattern as L={1,2,…,M}, where *M* stands for the number of light planes in the structured light pattern (M=11 in our case), and Index 1 represents the light plane that is closest to the bottom of *I*. The correspondence (or labeling) problem can then be defined as the mapping *ψ* that assigns each pixel segment from P an index (or label) from L:

(1) $$\psi :p_{i}\to L,\;\mathrm{for}\;i=1,2,...,N.$$

### 4.2. Labeling with Graphical Models

We follow the ideas presented in [32,33] and formulate the correspondence problem as an inference problem over probabilistic graphical models (PGMs). The formalism associated with PGMs allows us to break down complex problems into (smaller) simpler parts that can easily be modeled. For the labeling problem in the ATRIS sensor, these simpler parts correspond to geometrical relationships between pixel segments and their relative positions in a series of consecutive frames (captured by our ATRIS sensor).

Graphical models G are defined by a set of vertices V and a set of edges E connecting the vertices; that is, G=(V,E). To represent the labeling problem in the form of a graphical model, the pixel segments in the detected pattern are represented as vertices v∈V, and the dependencies between the pixel segments are represented as edges e∈E of the graph. Each pixel segment (and in turn each vertex) is associated with a discrete random variable *X* from $X^{t}=\left\{X_{1}^{t},X_{2}^{t},...,X_{N}^{t}\right\}$, where the set of all *N* random variables $X^{t}$ is defined by the binary distortion map *I* taken at time instance *t*. Similarly, each edge is associated with a factor that models the functional relationship between the vertices (random variables) connected by the edge. Solving the labeling problem defined in Equation (1) amounts to finding the most likely value (from L) for each random variable in $X^{t}$ given the dependencies (and relationships) between the pixel segments.

**Figure 5:**
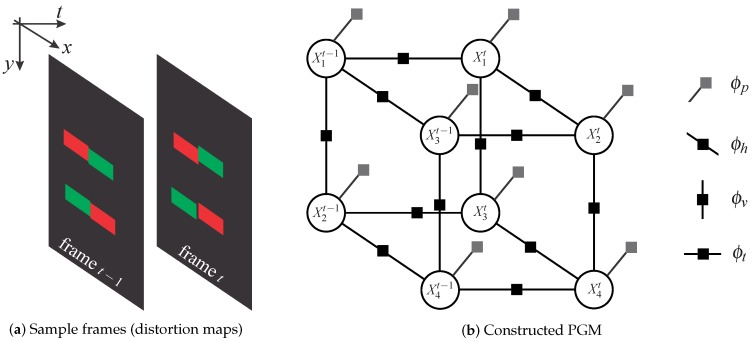
Illustration of the probabilistic graphical model (PGM)-based modeling procedure: (**a**) simplified distortion map; (**b**) corresponding PGM.

The PGM-modeling procedure used for the ATRIS sensor is illustrated in Figure 5. Here, the left side of Figure 5 depicts two sample frames, each containing two line fragments and a total of four pixel segments. The two frames are assumed to have been captured at two consecutive time instances, t−1 and *t*, and the color-coded pixel segments are assumed to be reasonably well aligned in the vertical (*y* axis), horizontal (*x* axis) and “temporal” (*t* axis) directions. The right side of Figure 5 shows the corresponding PGM constructed based on the two frames. As can be seen, the state (or value) of each random variable (i.e., each pixel segment) depends on the state of its horizontal, vertical and temporal neighbors. The dependencies between the neighboring pixel segments are defined by so-called factors (illustrated by squares), which model the relationships/dependencies between random variables and are for the case of horizontal, vertical and temporal neighbors denoted as $\phi_{h}$, $\phi_{v}$ and $\phi_{t}$, respectively. So-called unary factors are also used in our modeling procedure to construct the graph. These factors act only on a single variable (vertex) at a time and, in our case, encode the prior knowledge about the structure of the projected pattern [33]. They are denoted as $\phi_{p}$ in Figure 5.

With the illustrated modeling approach, the joint probability distribution of the PGM used in the ATRIS sensor can be written as a factor product:

(2) $$\begin{array}{ccc} p(X^{t-1},X^{t}) & = & \frac{1}{Z}\prod_{\begin{array}{c} t^{\prime }=t-1\\ \left(i,j\right)\in E_{h} \end{array}}^{t}\phi_{h}\left(X_{i}^{t^{\prime }},X_{j}^{t^{\prime }}\right)\prod_{\begin{array}{c} t^{\prime }=t-1\\ \left(i,j\right)\in E_{v} \end{array}}^{t}\phi_{v}\left(X_{i}^{t^{\prime }},X_{j}^{t^{\prime }}\right)\prod_{\left(i,j\right)\in E_{t}}\phi_{t}\left(X_{i}^{t-1},X_{j}^{t}\right)\prod_{\begin{array}{c} t^{\prime }=t-1\\ i=1 \end{array}}^{\begin{array}{c} t,N \end{array}}\phi_{p}\left(X_{i}^{t^{\prime }}\right), \end{array}$$

where *Z* denotes the partitioning function and the sets $E_{h}$, $E_{v}$ and $E_{t}$ correspond to subsets of all edges E, over which the horizontal, vertical and temporal factors are defined, respectively. In the above equation, *N* can in general also take different values at different time instances. The joint distribution is defined only for the case of two consecutive frames (from time instances *t* and t−1), but the extension to a longer sequence is trivial [33].

Inference over the constructed model can be conducted using various inference algorithms (e.g., [38] or [39]), where the goal is to find the most likely value (label) for each random variable in the constructed graph. A detailed description of the inference algorithm used for the ATRIS sensor is given in Section 4.2.3.

#### 4.2.1. Graph Construction

Unlike the toy example in Figure 5, where all line fragments are more or less parallel and the pixel segments are near perfectly aligned in all directions, building a PGM from real sensory data is a more complex task. Because no specific topology (e.g., nodes arranged in a grid) is present in the light pattern that is projected onto the target scene with the ATRIS sensor, it is necessary to formulate criteria for identifying vertical, horizontal and temporal neighbors. Based on these criteria, dependencies (i.e., factors) between neighboring pixel segments can be defined, and inference over the constructed graph can be conducted.

**Figure 6:**
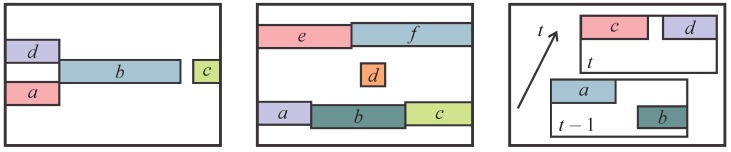
Defining the neighbors: horizontal neighbors (left image), valid neighbors are a-b, d-b; vertical neighbors (middle image), valid neighbors are a-e, b-e, b-d, b-f, c-f, d-f; (right image) temporal neighbors, valid neighbors are a-c.

For the ATRIS graph construction procedure, horizontal neighbors are defined as connected pixel segments (here, eight-adjacency is used [40] to probe for the connectivity). On the left side of Figure 6, where four pixel segments (labeled a, b, c and d) are presented, only segment pairs a-b and d-b represent horizontal neighbors, whereas the segment pair b-c does not, because b and c are not connected. The main motivation for introducing horizontal neighbors in the PGM construction procedure is to “encourage” horizontally-connected pixel segments to take the same label.

Vertical neighbors in the ATRIS sensor are defined as pairs of pixel segments that are not connected, but share at least one pixel at the same *x*-coordinate (and different *y*-coordinates). In the middle image of Figure 6, pixel-segment pairs a-e, b-e, b-d, b-f, c-f and d-f represent vertical neighbors according to this definition. Note that a pixel segment can easily have several vertical neighbors. Vertical neighbors are needed in the PGM to ensure that the detected light planes tend to be labeled consecutively. Due to this fact, vertical dependencies between pixel segments are extremely important for the modeling procedure.

Finally, temporal neighbors are defined as pixel segments belonging to detected patterns recorded at two consecutive time instances, t−1 and *t*, that share at least one non-zero pixel at the same spatial coordinates. This definition requires no tracking of the pixel segments over time and is extremely simple to implement. On the right side of Figure 6, only pixel segments a and c represent temporal neighbors, whereas all other segment pairs do not. Temporal neighbors are included in the graphical model to exploit additional temporal information when labeling the light planes of the projected pattern. As shown in the experimental section, the addition of temporal neighbors contributes to the accuracy of the labeling procedure.

The definitions presented define the topology of the PGM (i.e., vertices and edges) constructed from the given input image *I*. However, to be able to conduct inference on the graph, factors between pairs of neighboring vertices (or on a single vertex) that model the dependencies between the random variables associated with the vertices need to be defined, as well. The procedure for defining the factors used in this paper is described in the next section.

#### 4.2.2. Factor Definition

Factors represent functions of random variables. Typically, factors model the dependencies (relationships, constraints) between neighboring vertices and, hence, represent functions of two random variables. Alternatively, they relate only to a single vertex and act as functions of a single random variable. In the modeling procedure used for the ATRIS sensor, factors are used to model the relationships between horizontally-, vertically- and temporally-neighboring pixel segments, and unary factors are added to include knowledge about the structure of the projected light pattern.

Horizontal factors $\phi_{h}$ describe the relationship between horizontally-neighboring pixel segments and return a fitness score with respect to the labels assigned to the neighboring segments. The factor returns a score of one when both pixel segments are assigned the same label and some small score $f_{c}$ if they are assigned different labels. This definition reflects the structure of the projected pattern and encourages horizontal neighbors to take the same light plane label [33]. The fitness score returned by the horizontal factor is defined as:

(3) $$\phi_{h}\left(X_{i}^{t}=k,X_{j}^{t}=k^{\prime }\right)=\left\{\begin{array}{cc} 1, & k=k^{\prime },\\ f_{c}, & \mathrm{else} \end{array},\right.$$

where $k,k^{\prime }\in L$ and $f_{c}$ ($0<f_{c}<1$) denotes the fraction-cost parameter that penalizes horizontal neighbors that are labeled differently.

Vertical factors $\phi_{v}$ are assigned between random variables identified as vertical neighbors. The factor returns a high fitness score when the two vertically-neighboring pixel segments are labeled in an ascending manner and a fitness score of zero otherwise. This definition encourages the vertical neighbors to take consecutive light plane labels and prevents the assignment of labels in a non-ascending order. The relationship between vertically-neighboring segments in the ATRIS sensor is modeled as follows:

(4) $$\phi_{v}\left(X_{i}^{t}=k,X_{j}^{t}=k^{\prime }\right)=\left\{\begin{array}{cc} f(k-k^{\prime }), & k>k^{\prime }\\ 0, & \mathrm{else} \end{array},\right.$$

where $k,k^{\prime }\in L$ and *f* denotes a linear function of the difference of two labels. The function *f* decreases monotonically with the label difference:

(5) $$f\left(\delta \right)=\left\{\begin{array}{cc} g(1-(\delta -1)h), & \delta \neq 0\\ o_{c}, & \mathrm{else} \end{array}.\right.$$

The parameter *h* defines the slope of the linear part of the function *f*; $o_{c}$ (overlap cost) stands for a parameter that penalizes vertical neighbors with the same variable value; and the function g(.) represents a function that truncates all negative values to zero.

Temporal factors $\phi_{t}$ are assigned between pixel segments identified as temporal neighbors in two consecutive frames (of distortion maps). Under the assumption of a sufficiently high frame rate, the spatial location of most pixel segments can be considered constant. Pixel segments originating from two consecutive frames having approximately the same spatial location should therefore be assigned the same light plane label. The temporal factors defined for our modeling approach are functions that assign a fitness score of one if the pixel segments are assigned the same label and a fitness score of zero if the labels differ; that is [33]:

(6) $$\phi_{t}\left(X_{i}^{t-1}=k,X_{j}^{t}=k^{\prime }\right)=\left\{\begin{array}{cc} 1, & k=k^{\prime }\\ 0, & \mathrm{else} \end{array},\right.$$

where $k,k^{\prime }\in L$.

**Figure 7:**
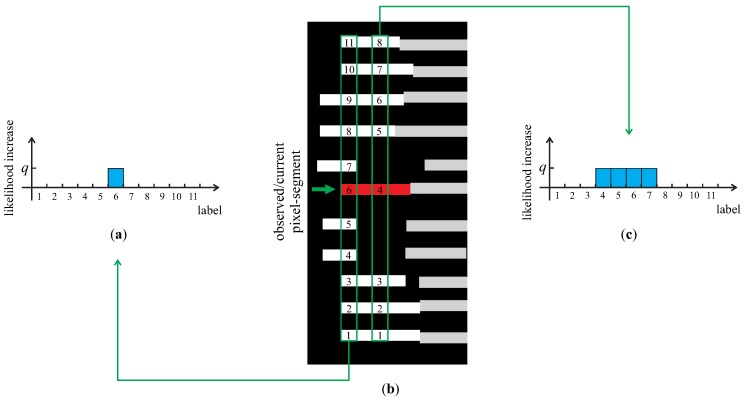
Computing prior factors: illustration of the procedure with a simple example (shown in (**b**)). For each random variable, the prior factor represents a probability distribution over all light-plane labels. Estimates of the probabilities are obtained by labeling the pixel segments and increasing the likelihood (shown in (**a**) and (**c**)) of the label assigned to the observed pixel segment at each *x*-coordinate.

Finally, the prior factors $\phi_{p}$ are assigned to all vertices and operate on a single random variable at a time. They are used to incorporate prior knowledge about the spatial structure of the projected pattern into the modeling procedure and in a sense carry information about the most likely range of values a random variable can take with respect to the vertical position of the pixel-segments and the number of its vertical neighbors above and below. The prior factors are computed based on the pseudo-procedure presented in Algorithm 1. Below, we outline the algorithm for a single pixel segment based on the toy example shown in Figure 7. However, the procedure is identical for all pixel segments.

Assume that our goal is to compute the prior factor for the red pixel segment and that all other pixel segments in image *I* are shown in white and gray (Figure 7b). To compute the prior factor, we first scan over all *x*-coordinates of the red segment and for each *x*-coordinate search for (at most *M*) line fragments above and below the current *x*-position of the red segment. We then label the pixel segments found at the current *x*-coordinate consecutively from the bottom of the image up and increase the likelihood of the label assigned to the red-segment by some arbitrary constant *q*. If we are able to find *M* pixel segments at the given *x*-coordinate, only the likelihood of a single label is increased (shown by the graph in Figure 7a), whereas the likelihood of several labels is increased if fewer than *M* pixel segments were found (shown by the graph in Figure 7c). The procedure aggregates the likelihoods over all *x*-coordinates of the red pixel segment and in the final step normalizes the likelihoods to the unit $L_{1}$ norm over all light plane labels to produce the final prior factor for the corresponding random variable.
**Algorithm 1** Calculating prior factors1:**for** all pixel-segments (i.e., random variables $X_{i}$) in the image *I*
**do**2: **Init:** Initialize p as an *M*-dimensional vector of all zeros3: **Result:** Normalized distribution (prior factor) $\phi_{p}\left(X_{i}\right)$4: **for** all *x*-coordinates of the pixel-segment corresponding to $X_{i}$
**do**5:  ▹ find (at most) *M* biggest line fragments in *I* having a pixel segment at the current *x*-coordinate6:  ▹ record the position, *k*, of the pixel-segment (corresponding to $X_{i}$) among the found *m* line fragments counting from the bottom of image *I* up7:  **if** the number of found line fragments *m* equals *M*
**then**8:   ▹ increase the *k*-th element of p by some positive constant *q*9:  **else**10:   ▹ increase all elements of p from position *k* to k+(M−m) by some positive constant *q*11:  **end**
**if**12: **end**
**for**13: ▹ normalize the vector p to unit $L_{1}$ norm; $\phi_{p}\left(X_{i}\right)=p$14:**end**
**for**

#### 4.2.3. Inference

To solve the labeling problem using the constructed PGM, a value needs to be assigned to each random variable (or vertex) constituting the graph, for which the range of possible values is given by the set of light plane labels L (see Equation (1)). The assignment is computed based on maximum a posteriori probability (MAP) estimation:

(7) $${\hat{X}}^{t}=\underset{X^{t}}{arg max}p\left(X^{t-1},X^{t}\right),$$

where $p(X^{t-1},X^{t})$ is the joint probability distribution of the PGM defined by Equation (2) and ${\hat{X}}^{t}$ is the most likely configuration of random-variable assignments for the PGM at time instance *t*.

MAP estimation can be conducted using different inference techniques; for example, [38,39,41]. In the case of acyclic graphs, an exact solution can be found; on the other hand, with cyclic graphs, as in our case, the problem is NP hard and only an approximate solution can be computed. Thus, for the ATRIS sensor, we use loopy-belief propagation for the inference on the PGM as described in [38].

## 5. Depth Image Reconstruction

In order to reconstruct a depth image of the target scene based on the labeled distortion map, we use a simple processing approach involving reference frames of the projected pattern taken at various distances from the camera.

**Figure 8:**
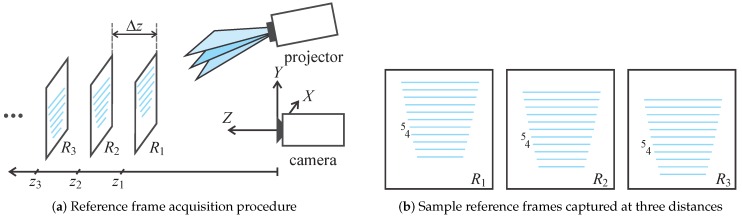
Illustration of the reference frame acquisition procedure: (**a**) the setup; (**b**) sample reference frames captured at distances $z_{1}$, $z_{2}$ and $z_{3}$. The reference frames are used to compute the depth value of each pixel segment in the labeled distortion map.

To capture the reference frames, we start by placing a planar surface parallel to the XY plane of the camera’s coordinate system (see Figure 8a) at some initial distance $z_{1}$ from the camera. We project our light pattern onto the surface and capture the first reference frame, $R_{1}$, corresponding to the distance $z_{1}$. We then move the surface by some depth increment Δz away from the camera and take another reference frame, $R_{2}$, at the distance $z_{2}$ from the camera. We repeat the procedure for the entire measurement range of our ATRIS sensor and thus generate a set of reference frames that are later used for depth calculation. The procedure for capturing the reference frames is illustrated in Figure 8a, and some sample frames are shown in Figure 8b. Here, the fourth and fifth light plane are labeled in each frame to demonstrate how the position of the detected light planes changes in accordance with the distance at which the reference frames are recorded.

Let us denote the distances at which the reference frames, $R_{s}$, were captured with:

(8) $$z_{s}=z_{0}+s\cdot \Delta z,\;\mathrm{for}\;s=1,\ldots ,S,$$

where $z_{0}$ denotes some minimum distance from the camera and the measurement range of the sensor lies between $z_{1}$ and $z_{S}$. The depth increment Δz defines the depth resolution of the ATRIS sensor and may be selected arbitrarily. Each reference frame, $R_{s}$, contains at most *M* line fragments $f_{i}^{\left(s\right)}$ (for i=1,…,m≤M) with associated light plane labels $k_{i}^{\left(s\right)}\in L$.

Consider a non-zero pixel (note that depth calculation is conducted for each pixel separately and not for the entire pixel segment at once) from the distortion map *I* located at image coordinates $p={\left[x_{pix},y_{pix}\right]}^{T}$ and associated with some light plane label k∈L assigned during the labeling procedure. To compute the ${\left[x,y,z\right]}^{T}$ position of the pixel (in camera coordinates), we first find the reference frame, $R_{\hat{s}}$, that contains the line fragment (at the same *x*-coordinate, i.e., $x_{pix}$) with the same label as the given non-zero pixel and is closest in terms of its *y* coordinate; that is:

(9) $$\hat{s}=\underset{s}{arg min}\left|y_{f_{i}^{\left(s\right)}}-y_{pix}\right|,\;\mathrm{subject}\;\mathrm{to}\;k=k_{i}^{\left(s\right)},$$

where $y_{f_{i}^{\left(s\right)}}$ stands for the *y*-coordinate of the *i*-th line fragment in the reference frame $R_{s}$. We then assign the distance, at which the frame $R_{\hat{s}}$ was taken, as the *z*-coordinate of the given pixel with respect to the camera’s coordinate system:

(10) $$z=z_{0}+\hat{s}\cdot \Delta z.$$

The *x* and *y* coordinates of the pixel (in camera coordinates) are computed using the hardware’s intrinsic parameters, which can be estimated with standard techniques [42]. The procedure presented is applied to all non-zero pixels of the spatial distortion map *I* and results in a sparse depth map, which is interpolated during the last processing step to fill in the missing values.

## 6. Experiments

This section describes the experiments conducted to demonstrate the merits of the developed sensor and to evaluate its performance on experimental data using quantitative performance metrics. We start the section by presenting experiments related to the characteristics of the sensor and the proposed acquisition procedure, proceed by providing results on the performance of the proposed light plane labeling technique and conclude the section with some examples of 3D reconstructions of scenes generated with our ATRIS sensor.

### 6.1. Characteristics of the Acquisition Procedure

**Figure 9:**
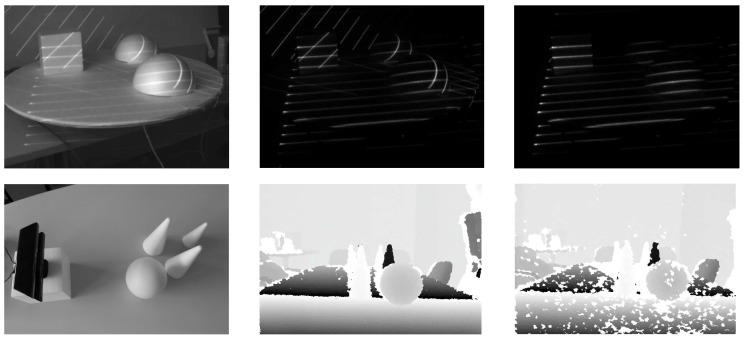
An illustrative example of the behavior of the developed acquisition procedure when two identical sensors are directed at the same scene and a comparison with a commercial sensor. Upper row: a sample scene illuminated with two ATRIS sensors (**left**); the demodulated images with non-cyclic orthogonal codes (**middle**); the demodulated images with cyclic orthogonal codes (**right**). Lower row: acquisition setup with two Kinect (v1) sensors (**left**); captured depth image when one sensor is active (**middle**); captured depth image when both sensors are active (**right**). Observe how the ATRIS sensor is able to compensate for the mutual interference, to recover a spatial distortion map and is unaffected by the pattern projected by the second ATRIS sensor.

One of the main merits of our pattern acquisition procedure is the fact that it is possible to deploy several depth sensors exploiting our procedure in the same environment. In fact, we demonstrated in [31] that it is possible to completely compensate for the mutual interference usually encountered when deploying several identical depth sensors in the same environment by constructing the modulation sequence of our acquisition procedure based on cyclic orthogonal (Walsh–Hadamard) codes. (A detailed discussion on the construction of the modulation sequence is beyond the scope of this paper. The reader is referred to [31] for detailed coverage of this topic.) An illustrative example of this characteristic is presented in Figure 9 on a simple indoor toy scene. Here, the images in the upper row correspond to our ATRIS sensor, and the images in the lower row correspond to images captured with the first generation Kinect sensor, which also exploits structured light [6]. The image in the upper left corner presents a sample scene with two of our sensors directed at it; the second image shows a demodulated image with non-cyclic orthogonal codes; and the last image in the upper right shows the demodulated image based on cyclic orthogonal codes. Note how the projected pattern can be recovered despite the presence of more than one active sensor operating on the same scene. In the lower row, the left most image depicts the acquisition setup using a pair of Kinect sensors. The middle image shows the depth map acquired when only one sensor is active, and the third image in the lower row demonstrates the effect of two Kinects capturing depth images of the same target scene. In the latter case, white areas appear in the image where depth information cannot be computed. This effect demonstrates the effect of the mutual interference of the two Kinects and is not present with the ATRIS sensor. As a consequence of the interaction of the Kinects’ light patterns, the shape of the objects comprising the scene is distorted, and part of the depth information is missing.

**Figure 10:**
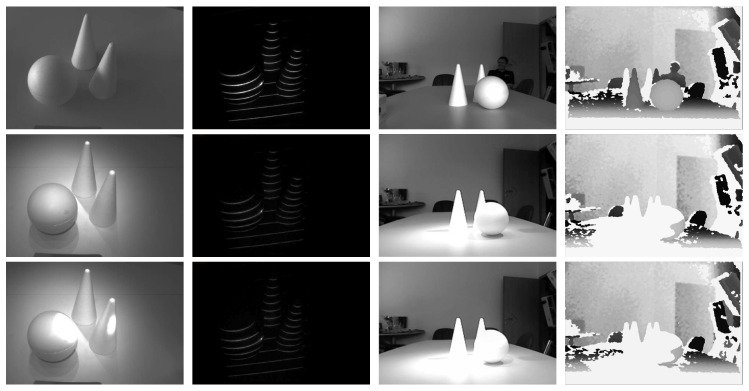
Qualitative examples of the performance of the developed acquisition procedure under various ambient lighting conditions. The first column shows a sample scene in different illumination conditions (from **top** to **bottom**): no additional illumination (**top**), with room lights on (**middle**) and with room lights on and a flashlight directed at the scene (**bottom**). The second column of images shows the distortion maps captured with the ATRIS sensor for the different illumination conditions. The third column depicts the same scene captured at the same time instance as the images in the first column, but with the Kinect sensor (v1). The last column presents the corresponding depth images generated by the Kinect sensor. Here, white areas indicate that no depth information could be computed.

Another important aspect of the developed pattern acquisition procedure is its robustness to ambient illumination and the presence of incident sunlight. To demonstrate this characteristic, we again provide a few (qualitative) illustrative examples. We first present sample results for a simple indoor scene imaged in three distinct illumination conditions: (i) under ambient lighting with no additional illumination directed at it (first row of Figure 10), (ii) under ambient lighting and with the room lights turned on (second row of Figure 10) and (iii) under ambient lighting, with room lights turned on and with a flashlight directed at the scene (third row of Figure 10). The first and third columns of Figure 10 show gray-scale images of the scene with the ATRIS prototype and Kinect sensor (taken at the same time instance), respectively, and the second and fourth columns show the corresponding distortion maps (for ATRIS) and depth images (for Kinect). Our acquisition procedure produces stable results with minor differences in the intensities of the distortion maps, but information is missing from the depth images generated by Kinect when the imaging conditions become more challenging. Note that missing information corresponds to white areas in the depth images.

**Figure 11:**
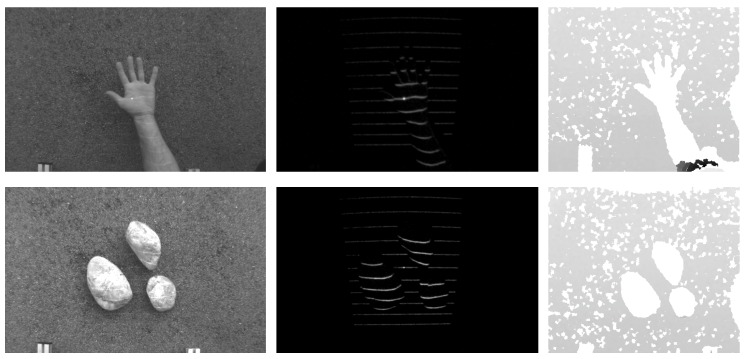
Illustrative example of the behavior of the developed acquisition procedure when deployed in outdoor environments. The first column shows gray-scale images of the target scene, the second column shows the distortion maps captured with the ATRIS sensor; and the last column of images presents the output of the Kinect sensor. The upper row presents images taken under exposure to moderate sunlight, and the lower row shows images taken under exposure to strong incident sunlight.

In our next experiment, we deploy our sensor outdoors and again provide comparative results with the first-generation Kinect sensor that uses the same imaging technology (i.e., active structured light) as our ATRIS sensor. The results of this experiment are shown in Figure 11. Here, the first column shows images of our scene with the projection pattern barely visible due to the incident sunlight; the second column shows images of the acquired distortion maps; and the third column shows images taken with the Kinect sensor. The upper row of images was taken under moderate incident sunlight, and the lower row of images was taken under relatively stronger sunlight. Note that the Kinect sensor is unable to acquire a complete depth map of the observed scene due to deployment outdoors (white areas in the output image of the Kinect sensor indicate that no data are available for that area), but our acquisition procedure produces stable (though noisy) distortion maps that can be used with our light plane labeling procedure. Although there are obvious differences in the number of pixels in which the two sensors interact with the scene, it is clear from the images presented that the ATRIS sensor is capable of operating outdoors in a robust manner.

**Figure 12:**
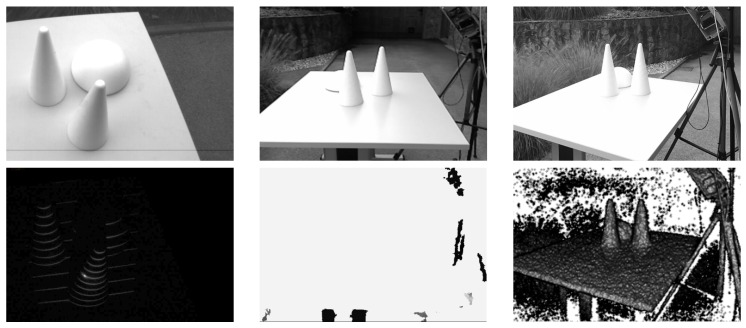
Qualitative comparison of the ATRIS sensor and both generations of the Kinect sensor on an outdoor scene. The first column shows the scene and distortion map from the perspective of the ATRIS sensor; the second column presents images from the first generation Kinect; and the last column of images shows the results produced by the second generation Kinect. Note that the second generation Kinect that uses time of flight technology, and our ATRIS sensor produces good results in all pixels measured, whereas the first generation Kinect performs less well outdoors.

For our third and last experiment with the acquisition procedure, we set up another outdoor scene and compared the performance of our ATRIS sensor with the first and second generation Kinect sensor. The second generation Kinect (v2) uses time of flight (ToF) technology and requires a full-fledged GPU supporting DirectX 11 to produce depth maps. Due to the required computing resources and different technology, Kinect v2 is not a direct competitor to our ATRIS sensor (which runs on a simple FPGA), but is included in our comparison to demonstrate the performance of a state-of-the-art depth sensor. The qualitative comparison is presented in Figure 12. Here, the first column of images represents the outdoor scene and corresponding distortion map captured with the ATRIS sensor; the second column depicts the scene and the depth image acquired with the first generation Kinect; and the third column shows the scene and the depth map captured with the second generation Kinect. As can be seen, both Kinect v2 and our ATRIS sensor produce solid, though noisy, results for all measured pixels, whereas the first generation Kinect struggles with its performance outdoors.

### 6.2. Characteristics of the Light-Plane Labeling Technique

The distortion maps (see Figure 9, Figure 10, Figure 11 and Figure 12) acquired with our ATRIS sensor form the basis for the light plane labeling procedure presented in Section 4. To evaluate the performance of the proposed procedure, we construct two datasets of spatial distortion maps.

The first dataset serves as our development set and is used in the experiments for tuning the open-hyper parameters of the labeling procedure (e.g., the values of $o_{c}$, $f_{c}$, *h*, etc.). In practice, it is necessary to fix the open-hyper parameters in such a way that the labeling technique exhibits the best possible performance. We therefore construct the first dataset from 152 images of a simple indoor scene, which is suitable for our purposes, because images taken indoors contain very little noise. The indoor scene comprises three objects positioned over a rotating table that change position backwards, forward, left and right, thus creating different depth discontinuities. The second dataset used for our experiments is a more realistic dataset of outdoor images. Here, we record 15 images of a scene containing a moving vehicle and a person passing between the vehicle and our ATRIS sensor. The images in this dataset contain objects with more complex geometry and are used to evaluate the performance of the labeling technique with fixed hyper-parameters.

All images from the two datasets are manually annotated to provide the ground truth for our experiments, in which we measure the accuracy of the labeling procedure using (what we refer to as) the correct labeling rate (CLR):

(11) $$\mathrm{CLR}=\frac{n_{c}}{N_{a}},$$

where $n_{c}$ denotes the number of correctly-labeled non-zero pixels and $N_{a}$ stands for the number of all non-zero pixels (the term “correctly” in this context stands for “being the same as the ground truth”). The correct labeling rate (CLR), as defined above, measures the fraction of correctly-labeled pixels among all pixels that have to be labeled. Note that the CLR in all graphs and tables presented below is computed over all images of the given dataset.

**Figure 13:**
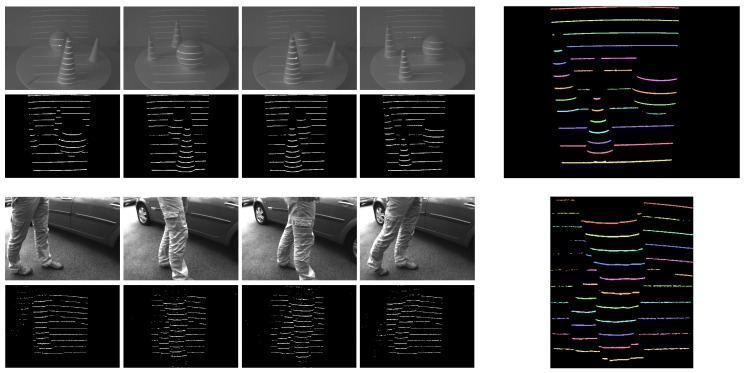
Sample images from the two datasets used in the experiments. The upper group of images shows sample images from the indoor dataset, and the lower group of images shows samples from the outdoor dataset. Both visible-spectrum and demodulated images are shown. The images on the right show the color-coded ground truth (best viewed in color).

A few sample images from both datasets and color-coded examples of the ground truth are shown in Figure 13. The upper group of images presents sample images from the (first) indoor dataset, and the lower group of images presents images from the (second) outdoor dataset. The imaging conditions outdoors are more challenging than the conditions indoors, which results in a higher level of noise in the demodulated images of the second dataset (observe the difference between the second and fourth row of images on the left). The images on the right side of Figure 13 represent annotated samples from both datasets.

As indicated above, the goal of the first series of experiments is to examine the impact of various hyper-parameters of the proposed techniques on the labeling accuracy. Towards this end, we first set the values of all hyper-parameters to a default value, then change a single parameter at a time and observe how the labeling accuracy changes with respect to the varying parameter. Even though the hyper-parameters of the proposed labeling technique are generally not mutually independent, we can, nevertheless, obtain a rough impression of the performance of the proposed method with respect to the varying parameter. We only make use of the first, the indoor dataset, in this series of experiments.

**Figure 14:**
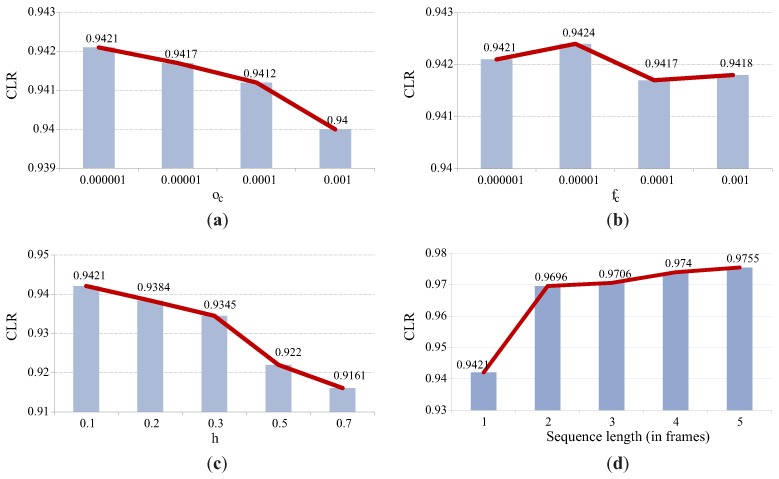
Impact of various hyper-parameters of the proposed technique on the labeling accuracy (measured in terms of the CLR). The results were generated on the indoor dataset and, except for the graph in the lower right corner, were computed using a sequence length of one. (**a**) impact of the overlap-cost parameter $o_{c}$, (**b**) impact of fraction-cost parameter $f_{c}$, (**c**) impact of the function drop rate *h*, (**d**) impact of the sequence length.

Figure 14 shows that the fraction cost $f_{c}$ (see Equation (3)) and overlap cost $o_{c}$ (see Equation (5)) have only a little effect on the labeling accuracy (graphs in the top row), whereas the function drop rate *h* (see Equation (5)), on the other hand, has a significantly larger impact on the labeling accuracy (graph in the lower left corner of Figure 14). These results suggest that the parameters $f_{c}$ and $o_{c}$ can be selected over a wide range of values with no significant performance loss, whereas *h* needs to be kept sufficiently small to ensure good performance. The most interesting observation of this series of experiments, which supports our working hypothesis that temporal information can improve labeling accuracy, can be made from the graph in the lower right corner of Figure 14. Note how the accuracy of the labeling procedure improves when two consecutive images from a sequence are used for constructing the PGM instead of only one. Adding additional images to the sequence further improves the labeling performance, albeit to a lesser extent. The best labeling accuracy we manage to achieve on the indoor dataset is a CLR of 0.9755 given a sequence length of five.

Based on the results of this series of experiments, the following parameter values are selected for the subsequent experiments on the outdoor dataset: $o_{c}=1e^{-6}$, $f_{c}=1e^{-5}$ and h=0.1. Note that it is not our goal to find values of the hyper-parameters that result in the best possible performance on the indoor dataset because this could lead to over-fitting and poor generalization abilities of the final labeling approach. We therefore make no further effort to find a better set of parameters for our technique and run a second series of experiments on the outdoor dataset with the hyper-parameter values listed above.

To gain insight into the characteristics of the proposed labeling approach and examine its behavior on more challenging data, we run several tests in the second series of experiments. These tests are conducted on the outdoor dataset and aim at: (i) examining the rationale behind defining the PGMs using horizontal, vertical and prior factors, (ii) evaluating the importance of temporal information on more challenging data and (iii) comparing the proposed technique to the existing labeling techniques.

**Figure 15:**
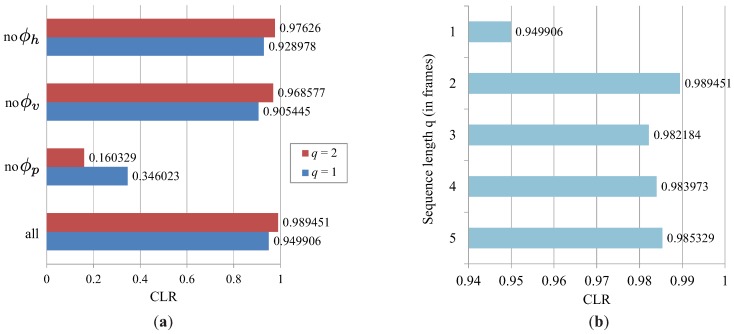
Results obtained on the outdoor dataset: (**a**) the results illustrate the importance of the selected structure of the PGM and (**b**) the impact of the sequence length on the correct labeling rate (CLR).

To demonstrate the importance of each of the factors in the PGM, we conduct several tests. During each test, we remove a single factor and keep the rest. (For example, we remove the horizontal factors $\phi_{h}$, which pull horizontally-neighboring pixel segments towards the same label, and keep only the vertical, prior and temporal factors. This case is denoted as “no $\phi_{h}$”. Other cases follow a similar notation.) We run the tests two times, first with a single image of the scene (i.e., q=1) and then with two consecutive images (i.e., q=2), to directly demonstrate the importance of the temporal factors, as well. The results of these tests are shown in Figure 15a. Several observations can be made from the results presented. First of all, the results indicate that prior factors are the most important component of the PGM. If the information about the structure of the projected patterns encoded in the prior factors is removed, the labeling accuracy drops significantly, to a value of CLR =0.346 (in the case of a single image), as shown by the graph labeled ”no $\phi_{p}$”. Without the prior information, the labeling accuracy becomes even worse when a second image is added to the sequence; that is, when temporal factors are introduced. However, when the prior factors are considered during the construction of the PGM, temporal information always improves the labeling performance. Similarly, both the horizontal and vertical factors also add to the overall labeling accuracy, as noticeable from the graphs labeled “no $\phi_{h}$”, “no $\phi_{v}$” and “all”. Here, “all” stands for the case when all four factor types are considered. All in all, the results of these tests suggest that all factors are important for the labeling procedure and contribute to the overall performance of the proposed labeling technique.

The graphs in Figure 15b show how the number of consecutive images used for constructing the PGM affects the labeling accuracy. As can be seen, the biggest increase in performance is noticeable when two consecutive images are used during construction of the PGM instead of one. The performance jump here is in fact a little larger than in the case of the indoor dataset, which can be attributed to the fact that the acquired distortion maps from the outdoor dataset are noisier, and hence, using more than one image from a sequence helps reduce the noise and determine the right labels. Interestingly, using more than two images from a sequence does not increase the performance further, but keeps it more or less stable. These results suggest that temporal information is useful for the labeling technique.

Next, we demonstrate the performance of the proposed labeling technique in comparison with other techniques that can be used for labeling the structured light pattern used in the ATRIS sensor. The implemented reference techniques are related to other structured light approaches from the literature that exploit light patterns comprised of parallel stripes (e.g., [43,44,45,46]), but, differently from these techniques, do not rely on coding strategies (in terms of color, intensity, geometry or time; see [18] for information on existing coding strategies) to solve the correspondence problem. Our comparison is therefore limited to techniques capable of handling uncoded structured light. Specifically, we implement the following reference techniques and include them in our comparison presented in Table 1:

- The naive labeling approach (NLA), which assigns light plane labels to the detected non-zero pixels in a consecutive manner. The first non-zero pixel at the given *x*-coordinate (looking from the bottom of the image up) is assigned the label 1; the second detected non-zero pixel at the given *x*-coordinate is assigned the label 2, and so on; until all 11 labels have been assigned.
- The labeling approach based on prior information (PR), which assigns light plane labels to the detected non-zero pixels by constructing a PGM based on prior factors only. This approach represents a refined version of the naive labeling technique introduced above.
- The reference approach from Ulusoy et al. (RUL) [32], which also exploits probabilistic graphical models, but relies only on spatial information to assign light plane labels to the detected non-zero pixels in the distortion map.

Note that even the naive labeling approach results in a relatively high labeling accuracy with a CLR of 0.888. This approach is expected to work well in simple conditions, where there is no noise in the detected distortion maps and no large depth discontinuities are present in the scene. All other techniques improve the performance of the NLA technique with the proposed approach resulting in a CLR of 0.989.

**Figure 16:**
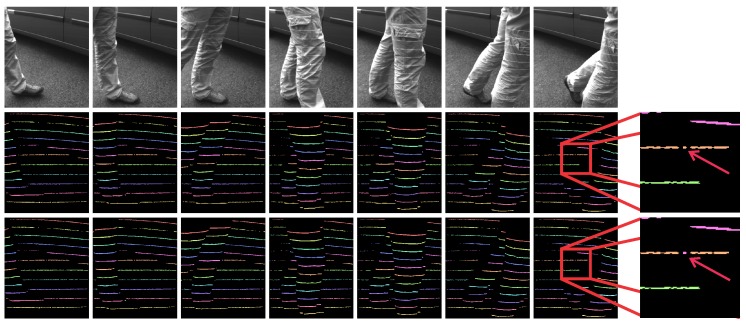
Visual examples of the results of the proposed light plane labeling procedure. The first row shows sample images from the outdoor dataset; the second row shows the (color-coded) manually-annotated ground truth; and the third row shows color-coded results of the labeling procedure. The images on the right side show an example of a labeling error, which is highlighted by arrows (best viewed in color).

All in all, the results of our experimental assessments suggest that exploiting spatio-temporal information for determining light plane labels in our ATRIS sensor is a feasible approach that results in state-of-the-art performance. The PGM approach is capable of assigning the correct label to most pixel segments of the detected light pattern even if large depth discontinuities are present in the scene observed. To visually demonstrate the efficacy of our approach, a few illustrative results of the labeling procedure are presented in Figure 16. Here, the first row depicts sample images from the outdoor dataset; the second row shows the color-coded ground truth; and the third row shows the color-coded results of the labeling procedure. Note how most of the assigned labels correspond to the ground truth, while there are, of course, a few errors, as well (right side of the image: the errors are marked with arrows). These errors typically introduce artifacts in the reconstructed depth images, but can easily be removed through simple post-processing of the depth images if they are not too frequent.

### 6.3. Constructing Depth Maps: 3D Reconstruction

**Figure 17:**
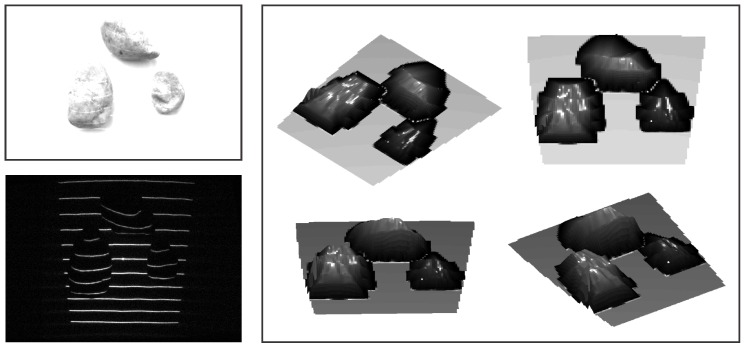
A visual example of the 3D reconstruction capabilities of the developed sensor. The image in the upper left corner shows a (visible light) image of the observed scene captured under strong incident sunlight; the image in the lower left corner shows the image of the detected light pattern; and the image on the right shows the reconstructed depth image from various angles.

Once the light plane labels have been assigned to all parts of the detected light pattern, depth images of the observed scene can be reconstructed using the procedure presented in Section 5. To demonstrate the result of this process for our ATRIS sensor, we again present a couple of illustrative examples. The first example, which is shown in Figure 17, shows a number of stones with relatively simple geometry. The stones were placed on a street outdoors, and an image was captured using a commercial camera (upper left corner of Figure 17), as well as our sensor. Note that, despite the exposure to relatively strong incident sunlight, the sensor was able to capture an image with the projected pattern clearly visible (lower left corner of Figure 17) and reconstruct the depth images quite well (right side of Figure 17).

**Figure 18:**
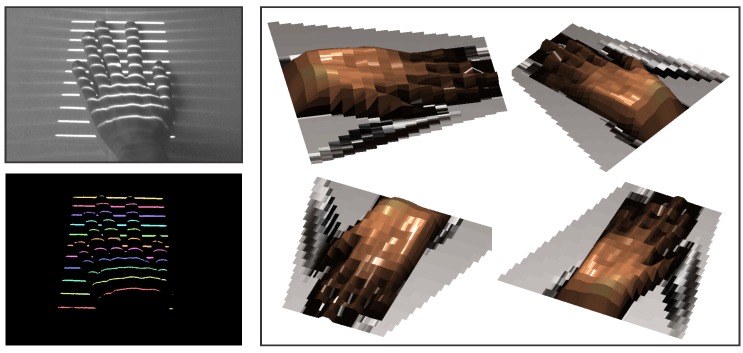
A visual example of the 3D reconstruction capabilities of the developed sensor. The image in the upper left corner shows a (visible light) image of the observed scene (i.e., a hand); the image in the lower left corner shows the labeled distortion map; and the image on the right shows the depth image from our ATRIS sensor from various viewing angles.

The second example in Figure 18 shows a gray-scale image of a hand captured with a commercial camera (upper left corner of Figure 18), the labeled spatial distortion map generated by our ATRIS sensor (lower left corner of Figure 18) and the 3D reconstruction from various viewing angles (right side of Figure 18). Note that, despite the more challenging geometry of the hand (compared to the stones in the first example), our ATRIS sensor successfully captures all parts of the hand and recovers a good-quality depth image. The results show that usable depth images can be obtained with our sensor in difficult imaging conditions, as well as with relatively complex geometry of the target scene. This makes the sensor applicable in outdoor applications that require reliable depth information regardless of the external imaging conditions.

## 7. Conclusions and Future Work

We have presented and experimentally demonstrated the merits of a novel sensor for depth image acquisition. The sensor presented is based on the recently-introduced concept of modulated pattern projection [31], which ensures that the procedure of detecting the projected light pattern is robust with respect to various factors, such as background noise, background illumination or the mutual inference of similar systems operating on the same scene. The procedure for determining the correspondence between the projected and detected light patterns, which forms the basis for depth image reconstruction, is implemented with an approach based on probabilistic graphical models and, in addition to spatial information, also exploits temporal information when solving the correspondence problem. As demonstrated in the experimental section, the proposed procedure performs well even when large depth discontinuities are present in the scene. The experimental results also show that the sensor presented is capable of acquiring stable distortion maps when competing commercial systems struggle with their performance.

As part of our future work, we plan to further improve the sensor presented. One of the main drawbacks of the current implementation is the structure of the projected light pattern, which affects the resolution and quality of the acquired depth image. To address this issue, we intend to explore structured light patterns that can be used with the PGM-based labeling procedure presented. The goal here is to devise a pattern that ensures an even better quality of the captured depth images compared to what is possible with the current sensor. A possible way to achieve this is by using more lines in the light pattern or combining the existing pattern with line scanning techniques capable of generating dense depth maps.

The current implementation of the ATRIS sensor is suitable for outdoor applications, such as collision avoidance or autonomous navigation, where approximate depth maps need to be acquired as reliably as possible and the resolution of the depth maps is not of major concern. Another application domain for our ATRIS sensor is computer vision applications exploiting action recognition [47,48], pose estimation [49,50], facial expression recognition [51,52] or motion analysis technology [53]. These applications are commonly deployed outdoors and could benefit from robust depth imaging technology.

## Figures and Tables

**Table 1:** Quantitative comparison with other labeling techniques (higher is better; 1 indicates a perfect score). NLA, naive labeling approach; PR, prior information approach; RUL, reference approach from Ulusoy et al.

Method	Outdoor Dataset (Noisy)			
	NLA	PR	RUL	Ours
CLR	0.888	0.912	0.950	0.989
